# COVID-19 in the UK and Occupational Health and Safety: Predictable not Inevitable Failures by Government, and Trade Union and Nongovernmental Organization Responses

**DOI:** 10.1177/1048291120929763

**Published:** 2020-05-25

**Authors:** Andrew Watterson

**Affiliations:** 1 Faculty of Health, University of Stirling, Stirling, UK

**Keywords:** COVID-19, UK, hazards

## Abstract

This commentary examines the occupational health and safety issues faced by the UK workers in the COVID-19 pandemic, against the background of government cuts in health care and in occupational health and safety budgets, and a deregulatory climate. The UK government has been obsessed, blinkered, and distracted by the desire to leave the European Union (Brexit). The state of knowledge about the virus, especially from international agencies that identified pandemic threats and strategies to combat it, is outlined. UK politicians, government bodies, medical and scientific advisors, and employers periodically ignored or abused that knowledge. Regulatory and ministerial inaction and errors on the workplace virus risks emerged. In contrast, several trade unions, health professional bodies, and nongovernmental organizations identified COVID-19 threats from poor personal protection equipment, working practices, and knowledge gaps and offered solutions for health care workers, social care workers, production workers, and service workers in “essential” occupations.

## Introduction

A combination of missed opportunities by government, their health and safety and public health agencies, and their advisors who ignored the COVID-19 pandemic warning signs has led to the UK possibly having the greatest mortality from the disease in Europe and the deaths and illnesses of many workers. An analysis of the information available in the UK on pandemics, the UK COVID-19 failures, and trade union and nongovernmental organization (NGO) actions was prepared for the Hazards Campaign, a UK-wide NGO that works to improve health and safety with trade unions and community groups, early in April 2020.^1^ This commentary is based on that analysis of the COVID-19 pandemic and focusses on the role of international agencies, the trade unions, and NGOs and to a lesser extent the UK government and its agencies as well as various NGOs.

**Figure 1.**
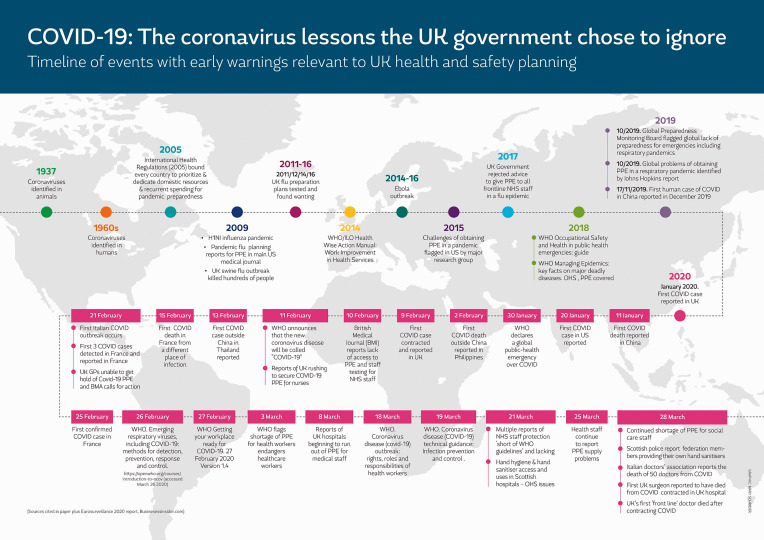
History of pandemics and coronavirus 1937 through end of March 2020.

It is vital to protect the health and safety of health and social care professionals, emergency workers, key support workers, and service and other workers at risk from COVID-19. Workers in the social care sector, service workers, and others were neglected in initial responses to the pandemic despite evidence and early warnings from China, South Korea, and Italy about the risks to these groups beyond the health care settings. The threat to UK workers’ health and safety has not been theoretical since December 2019. The UK government and devolved administrations in Wales and Scotland at the beginning frequently ignored both early warning and guidance on how to contain a pandemic from bodies such as the World Health Organization (WHO) and the International Labour Organization (ILO) and chose not to act on the faults identified in domestic pandemic preparedness exercises. These were not new pandemic lessons, as shown in the infographic (Figure 1). Generally applicable solutions to preventing risk from pandemic hazards have been known and advocated in global and national agencies and by NGOs for many years.

The novel coronavirus that causes COVID-19 is named SARS-CoV-2. COVID-19 has similarities and differences with influenza. Both viruses cause respiratory disease but there are important differences between the two viruses and how they spread. Both are transmitted by contact, droplets, and fomites,^2–6^ but SARS-CoV-2 transmission is also by aerosols that may travel beyond confined spaces.^4^ With mortality rates higher for COVID-19 than flu, the importance of having effective health and safety measures becomes even greater. SARS-CoV-2 transmission may occur by multiple routes. Research from China showed the virus could be transmitted through the touching of contaminated surfaces, viral aerosolization in a confined space, and contact with infected people who had no COVID-19 symptoms.^2^ Knowledge of these routes should have informed decisions weeks ago in the UK about occupational health and safety precautions, availability of sanitizers, what personal protection equipment (PPE) was needed, by whom, and in what settings. Information from Italy has provided further clues about the occupational health and safety risks run not just by health and social workers but many other key workers such as the police and support workers in different settings.

Many of the general precautions needed to protect workers from pandemics like COVID-19 were also identified after the flu pandemics in the 2000s and by the time of the Ebola outbreak in 2018 in the Democratic Republic of the Congo. There has been a widespread global recognition that a pandemic like COVID-19 would emerge but precisely what it would entail and when it would happen was not known. Although there have been multiple global problems and global failures in dealing with pandemics, important advice from international bodies and important lessons from other countries in the frontline of the COVID-19 epidemic have still been ignored and decisive action delayed by countries within the UK for reasons that are currently not entirely clear. UK readiness has been claimed by politicians but never evidenced.

The UK pandemic has occurred against a background of years of cuts in the health and social care sectors, public health, and decades of cuts specifically in the occupational and environmental health and safety agencies. Both sets of cuts have contributed to significant current worker as well as patient health and safety threats. Trade Unions and NGOs along with professional bodies and the medical press raised many concerns over decades about these cuts along with specific problems with PPE supply, use, suitability, and replacement for health care and other workers over several months. These problems have not been resolved by the UK government which all too often deflects and ignores them.

There have been suggestions in the UK that we are “all in this together” and that no one is responsible for the pandemic impacts and related health and safety failings. Both statements are untrue. The distribution of risks from COVID-19 is not equal across UK society. In addition to the high risks faced by health and social care professionals in acute, primary care and community settings, evidence is emerging for example that low-paid women in the UK are also at high risk of COVID-19 exposure^7^ along with low-paid workers elsewhere in our gig economy. The UK government has called for all its citizens to act responsibly to stem the mortality from the pandemic while its politicians and scientific servants have not, sometimes displaying willful ignorance if not criminal negligence about conditions faced by health care workers in hospitals and social care workers in nursing and care homes dealing with COVID-19 cases sometimes with no or insufficient and inadequate PPE.

In 2009 and 2015, researchers looked at the potential demand for and type of PPE needed, including respirators and surgical masks, during a hypothetical influenza pandemic.^8,9^ They were clear respirators were an important component of the infection control strategy and there were major logistical challenges in producing the numbers of respirators and masks needed.

## International Agencies

International agencies have a key role to play in disseminating information, but their information was either ignored or cherry-picked by UK politicians and scientists. These agencies themselves may be seriously under-staffed, under-funded, and under-resourced and therefore limited in what they can do. Nevertheless, they have still produced some valuable, comprehensive and practical advice about the best public health policies for nations to adopt when faced with pandemics. This was the case with COVID-19. If the UK had planned for the pandemic as WHO guides suggested and acted on WHO advice about testing, tracing, and isolating, then the public health and worker health and safety disaster that followed could have been reduced.

The WHO has been able to disseminate important COVID-19 information, based on its earlier work as well as the Chinese and South Korean experiences, to many other countries in 2020. Between 2018 and the UK pandemic outbreak, WHO identified a range of appropriate measures necessary to deal with pandemics involving planning and equipping health staff with suitable health and safety equipment. It even produced detailed manuals and guidelines for governments on how to manage pandemics and protect health workers in such pandemics.^11–17^ The WHO/World Bank Global Preparedness Monitoring Board reports from 2019 also raised the lack of global preparedness for a respiratory global pandemic. Issues around PPE for health workers were specifically touched up: a major UK failing in 2020. The 2019 reports were widely covered in the UK press. Yet UK COVID-19 practices fell far short of WHO guidance.

WHO COVID-19 pandemic guidance on the rights, roles and responsibilities of health workers indicated:

… health worker rights include that employers and managers in health facilities: assume overall responsibility to ensure that all necessary preventive and protective measures are taken to minimize occupational safety and health risks; provide information, instruction and training on occupational safety and health, including; refresher training on infection prevention and control (IPC); and use, putting on, taking off and disposal of personal protective equipment (PPE); provide adequate IPC and PPE supplies (masks, gloves, goggles, gowns, hand sanitizer, soap and water, cleaning supplies) in sufficient quantity to healthcare or other staff caring for suspected or confirmed COVID-19 patients. … consult with health workers on occupational safety and health aspects of their work and notify the labour inspectorate of cases of occupational diseases; not be required to return to a work situation where there is continuing or serious danger to life or health, until the employer has taken any necessary remedial action; allow workers to exercise the right to remove themselves from a work situation that they have reasonable justification to believe presents an imminent and serious danger to their life or health. When a health worker exercises this right, they shall be protected from any undue consequences.^15(^^pp^ ^1–2^^)^

Accounts from UK health professionals, paramedics, and emergency workers continue to reveal that many of these worker rights have never been observed by employers and no enforcement action taken by regulators.

The ILO, a tripartite body of employers, employees, and governments, has the global lead to produce conventions on working conditions including occupational health and safety and provides reports on these topics. The ILO in March 2020 found that unprotected workers, including the self-employed, casual and gig workers, were most likely to be disproportionately hit by the virus because of a lack of either paid or sick leave. Migrant workers were particularly vulnerable to COVID-19 impacts which limited their ability to access their places of work in destination countries and also to return to their families. In the UK, significant numbers of workers still fall into these groups.

The ILO was unequivocal in calling for policy responses that firstly made sure workers, employers, and their families were safeguarded from COVID-19 workplace health risks and community risks with large-scale public support and investment.^18^ The UK is a long way off in achieving these objectives.

## The UK Government

The policy of UK government on COVID-19 in April 2020 failed workers across the health care system, through social care, the emergency sector to transport workers, shop workers, and construction and refuse workers. The UK government determines the national health, social care and workplace health and safety policies, staffing, national purchasing and planning of services, and related infrastructure and other spending. Its priorities and projects provide both the frame and main engine within which we need to assess the impact of COVID-19 on our society. Successive governments have run down the National Health Service (NHS) and the budgets of regulators and implemented a deregulatory and reduced regulation approaches to workplace health and safety openly and also covertly.

Issues of NHS preparedness to deal with UK health needs, generally, and a pandemic, in particular, have long been raised. Problems including provision of PPE have occurred over the last four or five years.^19,20^ Significant problems relating to health and safety as well as public health protection emerged in the 2010s. Evidence that cost rather than public health and workers’ health and safety dominated decisions not to purchase PPE for health workers has emerged.^21^

England’s Deputy Chief Medical Officer, Dr Jenny Harries, said on March 20, 2020: “The country has a perfectly adequate supply of PPE.” The Lancet in contrast found that:

… examples daily of doctors having to assess patients with respiratory symptoms but who do so without the necessary PPE to complete their jobs safely. Health workers are challenged if they ask for face masks. Even where there is PPE, there may be no training. WHO standards are not being met. Proper testing of masks is being omitted. Stickers with new expiry dates are being put on PPE that expired in 2016. Doctors have been forced to go to hardware stores to buy their own face masks. Patients with suspected COVID-19 are mixing with non-COVID-19 patients.^22^

The rhetoric of UK ministers and scientific civil servants on protecting health care and other workers from COVID-19 has been strong but their policies and implementation have been woeful. As of April 23, 2020, some 119 NHS workers have died from COVID-19.

## The Health and Safety Executive

The Health and Safety Executive (HSE) enforces a range of UK legislation to protect the health and safety of workers but has simply gone missing on COVID-19. For months HSE appeared to do nothing to enforce serious breaches of health and safety law when a whole range of employees inside and outside the health care system were visibly at risk from non-availability of appropriate PPE and physical distance failures in the workplace. HSE covers hospital and local authority care homes and social care. Local authority-based inspectors may enforce health and safety laws in locations such as smaller workplaces and nursing homes.

HSE produces extensive information on the web and standard guides to managing health and safety and carrying out risk assessments.^23,24^ These laws and guidance should have ensured action on several easily remedied health and safety problems that emerged with COVID-19. What HSE has been doing, could do, should do, and will be doing to protect during the pandemic merits urgent investigation now. In response to a letter from the UK Hazards Campaign, the HSE, on April 2, 2020, confirmed what actions HSE had taken on the COVID-19 pandemic. It is not obvious the extent to which the HSE was consulted by the UK government and employers on COVID-19 health and safety at work impacts and controls. HSE’s role from the beginning was marginalized because it accepted the Department of Health and Social Care, working closely with Public Health England and the devolved administrations was the lead government department for the UK’s response. The health and safety of workers, so seriously under threat during and due to COVID-19, was effectively side-lined even before the pandemic gained pace.

On April 2, 2020 HSE circulated further COVID-19 information but stressed its policy would be “flexible and proportionate.”^25^ In a pandemic when health care and other workers’ lives are threatened, many would expect “proportionate” to mean great activity on COVID-19 in inspecting, monitoring, advising, and where necessary enforcing regulations against bad employers. This seems to have meant inaction again although NGOs pressed for and eventually got a “public” HSE hotline for COVID-19 enquiries about workplace hazards. By mid-April, there was further confusion about how HSE could and would ensure the reporting and investigation of illnesses and deaths of workers from COVID-19.

The advice of Public Health England on PPE and their various revisions of what was and was not required and for whom caused further confusion and controversy. The advice has at times been queried by clinicians in the field and revised at times for reasons that have not yet fully emerged but were considered to be lower PPE standards than those in WHO guidelines. Local authorities across the UK and their environmental health officers should have a role to play in addressing COVID-19 health and safety issues but have not been used extensively so far.

Employers have a duty to report occupational diseases, although COVID-19 is not yet classified as an occupational disease under the Prescribed Industrial Diseases scheme which would generate workers’ compensation.^26^ In contrast, countries like Denmark already recognize COVID-19 as an occupational disease.^27^ Where an incident at work has led to someone’s exposure or possible exposure to coronavirus, reports are made to HSE under The Reporting of Injuries, Diseases and Dangerous Occurrences Regulations 2013. Reports should also occur when a worker has been diagnosed with COVID-19 and there is reasonable evidence that it was caused by exposure at work and where a worker dies as a result of occupational exposure to coronavirus. There are concerns, however, that all occupational COVID-19 cases will not be recorded and reported and those that are may not be fully investigated. At a later date, there could be civil actions in the courts by employees who contracted COVID-19 at work or by their families if fatalities occurred.

## Trade Unions and Professional Bodies

Trade unions have worked much more effectively than the government regulators at raising occupational health and safety problems due to COVID-19. Trade unions framed many of their responses to workplace hazards in terms of the need for employees to have decent work and fair work. They viewed many of the COVID-19 risks as preventable. When employees are affected, trade unions work to ensure employment rights, job security, and access to sick pay and support to be protected. The unions have found flawed government, NHS, and some employer responses to COVID-19. They have been faced at times with run-down services, limited or nonexistent resources that have seriously impacted on the health and safety not just of front-line clinical staff but also workers in a wide range of other sectors and occupations.

The Trades Union Congress has produced a range of information on COVID-19 including planning advice, health and safety information, employment rights, and links to resources including standard government advice.^28^ It provided case studies on workplace hygiene and relevant health and safety laws along with detailed information on PPE and flagged the need for specific precautions relating to “public-facing workers.” The General, Municipal and Boilermakers Union (GMB) represents members for example in the health sector, social care, local authorities, manufacturing, and services where there are numerous COVID-19-related occupational health and safety issues. They advise and represent members in these locations and have produced a detailed briefing for members on COVID-19^29^ with information on the law and worker rights, on symptoms, transmission, precautions, and risks. There is specific information about what PPE should be supplied and what face masks in addition to other actions their employer should take to protect their health and safety.

GMB has catalogued many wholly avoidable health and safety problems that workers have faced with COVID-19. They have included access to PPE for hospital porters, lack of protective clothing and sanitizers for hospital workers, ambulance workers left with no hand sanitizers, wipes, and masks, faulty testing gear, airport staff with no gloves or sanitizers, and gig workers abandoned and penniless when faced with coronavirus threats. When unions like GMB raise concerns, they are frequently described as “unhelpful” by employers, a response that seems especially inappropriate in current circumstances. Unite, the trade union, also has members in a very wide range of sectors including health and public service, labs, construction, manufacturing, transport, and service industries. It has produced some of the most detailed COVID-19 guidance and checklists for trade unionists, open to all on its web pages. It covers for example those in clinical and nonclinical settings, critical workers.^30^

The emergency services—police, fire, paramedics, and ambulance—have a critical role to play during the pandemic. The Fire Brigades Union (FBU) has produced generic guidance on COVID-19 for its members^31^ and flagged the problems of the lack of testing needed to determine which of their members who are or have been self-isolating have COVID-19. Failure to test people for COVID-19 has seriously affected staffing levels in the brigades. This jeopardizes the health and safety of its members in operational settings. Similar problems will exist in the other emergency services. The Scottish Police Federation even set up its own panel to assess PPE it had been offered and found the equipment wanting.^32^

The BMA, the “doctors union,” the *British Medical Journal*, and *Lancet* raised numerous health and safety issues at an early stage relating to COVID-19 across the UK affecting health care and social care workers. They included serious PPE supply and suitability problems^33^ as well as issues with risk management, health and safety procedures, staffing, wider resource issues, staffing levels, and stress and fatigue.

The Royal College of Nursing, a trade union with a professional role too, had an ambitious plan and at the beginning called for:

… priority COVID-19 testing for all health care professionals, access to adequate supplies of personal protective equipment and hand sanitizer for all nursing, midwifery, social care and student nurse staff for use at the point of care, full occupational sick pay paid from day one for all our members, with no detriment, regardless of where they work. Provision from government and employers to ensure all nursing staff can care for their children without a loss of income. Clarity on the measures taken to protect pregnant and vulnerable nursing staff. Stringent measures in place to ensure the health, safety and wellbeing of staff by addressing fatigue, hydration and issues of abuse towards staff.^34^

By April 18, 2020, there was no sign in the UK that all PPE problems had been resolved in all areas and issues existed for pregnant staff working and childcare support.

The Society of Occupational Medicine (SOM), British Occupational Hygiene Society (BOHS), and Faculty of Occupational Medicine of Royal College of Physicians (FOM) have been active. FOM produced a variety of guidance materials on COVID-19 sometimes with other professional bodies and societies such as the BOHS and the SOM. SOM, BOHS, and other groups have pressed for COVID-19 testing of all key workers and have called for an investigation of the supply of suitable PPE for health professionals.^35^ By April 6, 2020, SOM spoke out along with other societies on COVID-19 with a strong and unequivocal statement exposing failures in UK occupational health and safety policy on COVID-19. It did not believe:

… work related fatalities due to COVID-19 exposure is a given. The UK should have aimed for a target of zero work caused fatalities in this pandemic within the NHS, essential services and UK business. With proper application of controls, no worker should die of work acquired COVID-19.^36^

## Nongovernmental organizations

Hazards Campaign is a group of activists and trade unionists that has a long history of campaigning for occupational health and safety in the UK. The campaign has provided excellent, accessible, and clear information on COVID-19 that is much more detailed than that available from many employers.^37^ The campaign knew of many of the workers with the greatest health and safety concerns who would be in workplaces on precarious contracts and not in a trade union-organized and supported workplace. In such workplaces, it would be impossible for these employees to resolve COVID-19 issues “through speaking with their employer or trade union” first. They would risk losing their jobs.

The campaign group raised major concerns with HSE about contradictory and flawed COVID-19 advice provided by the UK government. In particular, the group raised the impossibility of keeping safe distances in some workplaces and some jobs as well as contact of workers with materials. Sectors affected included manufacturing, construction, and warehousing, for example, involves a lot of touching and handling of materials. In many workplaces, complying with the hand washing, cleaning surfaces, and materials guidance would be impossible. The campaign asked HSE to provide details of their actions on COVID-19. Hazards magazine, working with the Hazards Campaign, has examined the occupational health and safety policies and practices of employers and government regulators again over many decades. It has produced an incisive, accessible, and readable analysis of the COVID-19 failures in the UK and extensive sources of information for workers on the pandemic from a health and safety perspective.^38^ The analysis stresses that the pandemic could persist “because public health was a low priority and workers did not have the sick pay and job protection necessary to survive.” It also details the need for PPE to protect workers worldwide from COVID-19 and describes some of the interventions from the WHO and International Trade Union Confederation (ITUC).

## Employers

Large- and medium-sized employers will have their own health and safety advisors as do bodies like the Confederation of British Industry and will have worked out plans for dealing with COVID-19. Much of bespoke employer information available focuses on economic and financial impacts of COVID-19 and not on the occupational health and safety threats to employees. How successful employer plans have been in protecting employees will be a matter for careful scrutiny after the pandemic has ended but already it is clear there have been major failings by some employers in protecting their staff from exposure to COVID-19 with extensive media footage of workplaces showing employees closely packed and lacking suitable protection and PPE where it should have been available. The CBI in UK has little specific information relevant to occupational health and safety on its COVID-19 web page although it has working groups looking at people redeployment, keeping the nation healthy, and supporting families in hardship which may impact on health and safety. In addition, it provides an assessment of what UK business can learn from the actions of the Chinese government to deal with COVID-19.^38,39^

## Conclusions

A catalogue has emerged in the UK of missed opportunities and failures by various government bodies, agencies and organizations, and employers to plan for the pandemic and to equip staff with the necessary health and safety equipment and procedures to protect themselves and the public from COVID-19. International agencies, foreign governments, researchers, UK trade unions, and NGOs in contrast did not simply provide early warning about the pandemic but offered important guidance on solutions to mitigate its impacts on workers and hence wider society. The unions drew on precautionary principles and evidence-based assessments of risks unlike the UK government which still appears to be drawing on policy-based evidence selection.

Many of the occupational health and safety pandemic threats in the UK are ongoing because of a lack of planning. Yet the UK plans decades ahead for military scenarios and spends billions of pounds on equipment and systems just in case of conflict. There is therefore no reason why governments cannot choose to plan ahead for pandemics and spend much smaller sums on equipping health care and other workers with the equipment and systems they need to protect the public which requires we protect their health and safety. We owe it them and the government owes it to us.

To date, there are no official UK-wide estimates for health care worker or any other group of workers’ morbidity and mortality from COVID-19 but efforts are being made to collect such data globally. There are no estimates for the number of service workers, transport workers, and manufacturing workers who have died from COVID-19 due to workplace or work-related exposures.

Now and in the future trade unions and NGOs want an active, properly resourced and staffed health and safety regulator not cowed or marginalized by government and employers. A pre-occupation with better regulation and running down regulatory capacity in areas critical to worker health and public health has proved disastrous in the pandemic. It should cease. HSE and other regulators in local authorities should have been checking, prior to the pandemic, pandemic health and safety planning, health care PPE and ensuring that PPE and health and safety procedures for social care staff and workers in social care, shops, warehouses, other workplaces, and transport were available, fit for purpose, and applied. It is not clear from information in the public domain that they did so.

Ensuring that all vulnerable and precarious workers in our gig economy have adequate economic support if they must stop work is essential. Not to provide it will increase the possibility that they will be at risk from continuing to work in hazardous settings because they need to obtain food and pay their bills. Governments and employers should support and facilitate the work of the trade unions and NGOs who have reached employees in many workplaces with some of the best and most up-to-date advice and support on COVID-19 occupational health and safety. It has been done on very limited budgets but with great effect.

In due course, there will need to be a thorough analysis of the national and regional performance of the UK and devolved governments during the pandemic, why some decisions and actions varied between them and with what effect on employee health and safety across society. Also the wisdom of the devolved administrations accepting initial UK government policy and agency assessments of pandemic risks should be scrutinized. The first UK timetable for actions rather than those of the WHO with its extensive evidence-based reports on pandemics was seriously flawed. The implications for occupational health and safety were considerable.
